# Albendazole-Induced Liver Injury Mimicking Autoimmune Hepatitis

**DOI:** 10.7759/cureus.114878

**Published:** 2026-08-20

**Authors:** Aren Nersisyan, Narek Kocharyan, Lusine Mardanyan, Arman Mirumyan, Arshak Shahenyan

**Affiliations:** 1 Department of Gastroenterology and Hepatology, Nairi Medical Center, Yerevan, ARM; 2 Department of Histopathology, Davidyants Polyclinics, Yerevan, ARM; 3 Department of Pathophysiology, Yerevan State Medical University, Yerevan, ARM

**Keywords:** albendazole antihelminthic, autoimmune liver disease, autoimmune vs drug-induced liver damage, drug-induced liver injury (dili), liver damage

## Abstract

Drug-induced liver injury is one of the leading causes of acute liver injury and may mimic other liver diseases, including idiopathic autoimmune hepatitis. Although albendazole is generally a safe antiparasitic agent, it can be associated with rare cases of clinically significant liver injury; it can even cause a mixture of both types of injuries, or a type of hepatitis that is similar to an autoimmune disease, such as positive autoantibodies and elevated IgG, making differential diagnosis more difficult. We present a case of albendazole-induced liver injury mimicking autoimmune hepatitis in a 41-year-old woman with marked hyperbilirubinemia, positive ANA, and elevated IgG levels, in whom liver biopsy and clinical follow-up played an important role in clarifying the diagnosis.

## Introduction

Drug-induced liver injury (DILI) is one of the most common causes of acute liver injury. It may present with a broad spectrum of clinical and histopathological manifestations ranging from asymptomatic elevation of liver enzymes to acute liver failure. Numerous medications and herbal supplements have been implicated in DILI, making diagnosis challenging.

Albendazole is a medication that is often used to treat parasitic infections. It's usually thought to be safe, but sometimes it can cause serious liver injury. There have been some cases reporting albendazole-induced liver injury. Albendazole is rapidly converted in the liver to the primary metabolite, albendazole sulfoxide, which is further metabolized to albendazole sulfone and other primary oxidative metabolites that have been identified in human urine [1]. Albendazole therapy has been associated with transient and asymptomatic elevations in serum aminotransferase levels in up to 50% of patients treated for more than a few weeks. These abnormalities rapidly improve with the cessation of therapy. Albendazole has also been associated with rare instances of clinically apparent liver injury. The likelihood score of albendazole is B (highly likely cause of clinically apparent liver injury) in LiverTox. The onset of injury has been within a few days to as long as two months from starting therapy but can be more rapid with multiple courses of treatment. The pattern of serum enzyme elevations is typically hepatocellular or mixed. Allergic features (rash, fever, eosinophilia) may be present but are not prominent [2].

Drug-induced autoimmune hepatitis represents a distinct type of DILI characterized by elevated transaminases, positive autoantibodies, hypergammaglobulinemia, and histological findings similar to idiopathic autoimmune hepatitis. Differentiation between idiopathic autoimmune hepatitis and drug-induced autoimmune hepatitis may be difficult [3].

We present a case of albendazole-induced liver injury mimicking autoimmune hepatitis in a 41-year-old woman with marked hyperbilirubinemia, positive ANA, and elevated IgG levels, in whom liver biopsy and clinical follow-up played an important role in clarifying the diagnosis.

## Case presentation

A 41-year-old woman presented to our clinic for a second opinion regarding her diagnosis. Six weeks before presentation, she took four tablets of albendazole 400mg and subsequently took an additional two tablets 10 days after the first time. Four weeks before presentation to our clinic, she presented to the infection clinic with complaints of generalized weakness, jaundice, nausea, and subfebrile temperature. All of these symptoms had started about a week before presentation to the infection clinic. She has a past medical history of osteopenia, for which she was taking vitamin D and calcium supplements. A gastroenterology consultation was performed. 

Additional autoimmune liver serologies were negative. The R ratio at presentation was 43.6, consistent with hepatocellular injury (Table 1).

**Table 1:** Laboratory Findings at Presentation to the Infection Clinic (Four Weeks before the Presentation) AST, aspartate aminotransferase; ALT, alanine aminotransferase; ALP, alkaline phosphatase; GGT, gamma-glutamyl transferase; INR, international normalized ratio; PT, prothrombin time; IgG, immunoglobulin G; ANA, antinuclear antibody; U/L, units per liter; µmol/L, micromoles per liter; g/L, grams per liter; sec, seconds.

Parameter	Result	Reference Range
Aspartate aminotransferase (AST), U/L	1950	10-40
Alanine aminotransferase (ALT), U/L	3440	7-56
Alkaline phosphatase (ALP), U/L	183.01	40-130
Gamma-glutamyl transferase (GGT), U/L	126.25	9-48
Total bilirubin, µmol/L	108	3-21
Direct bilirubin, µmol/L	74.5	0-7
International normalized ratio (INR)	1.51	0.8-1.2
Prothrombin time (PT), sec	20.8	11-14
Immunoglobulin G (IgG), g/L	22.44	5.52-16.31
Antinuclear antibody (ANA), titer	Positive (1:320)	Negative

The patient underwent a thorough investigation for infectious diseases, but all tests came back negative. These tests included checks for hepatitis A and E viruses, hepatitis B and C viruses, HIV, Epstein-Barr virus, Brucella, and syphilis. Additionally, measurements of ceruloplasmin and copper levels in the urine over 24 hours were all within normal ranges. Liver biopsy was performed.

Histopathological findings

**Figure 1:**
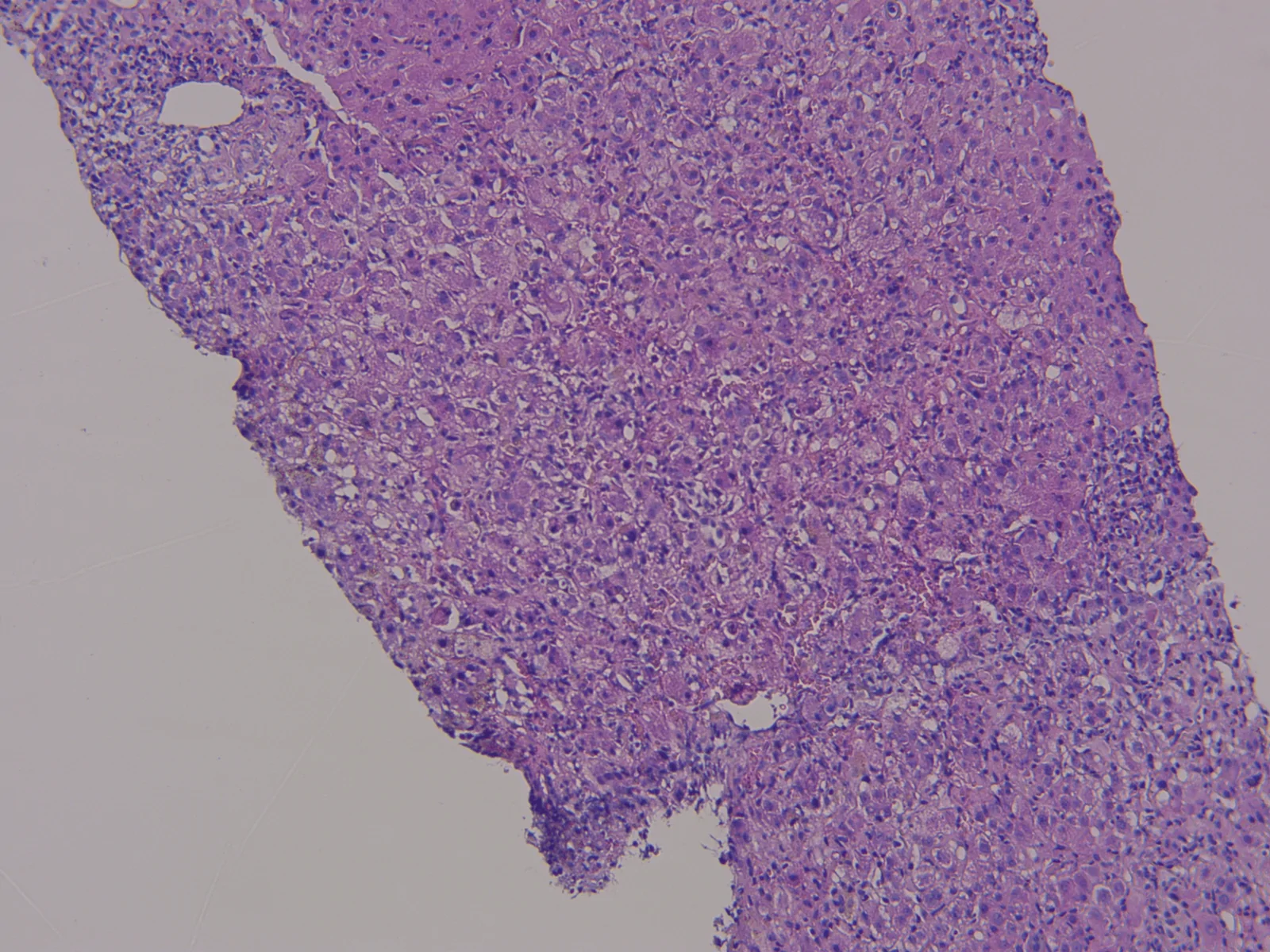
Hematoxylin and Eosin Staining X40. Intralobular mononuclear infiltration

**Figure 2:**
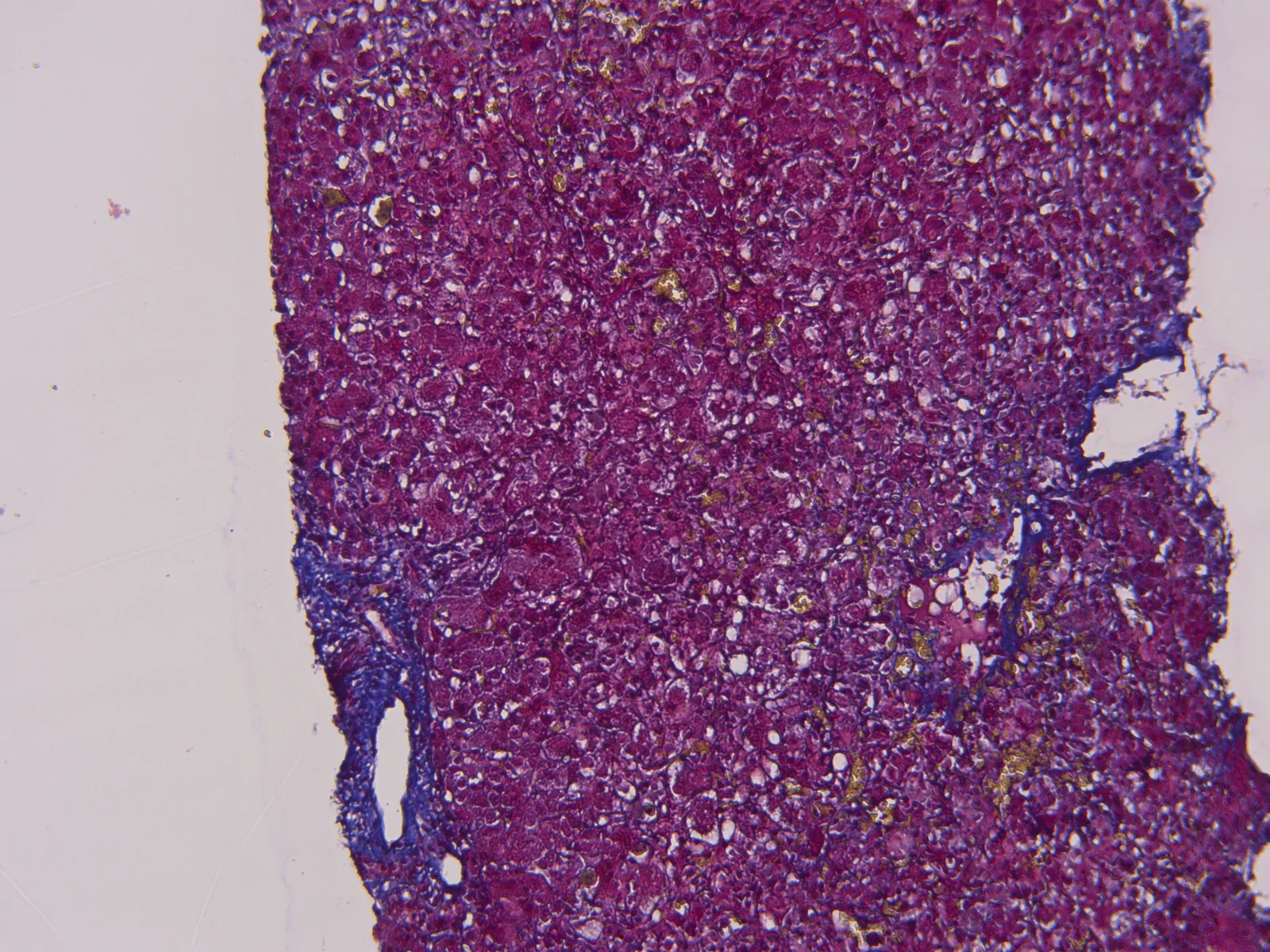
Masson Trichrome Staining X40. There was no significant fibrosis

**Figure 3:**
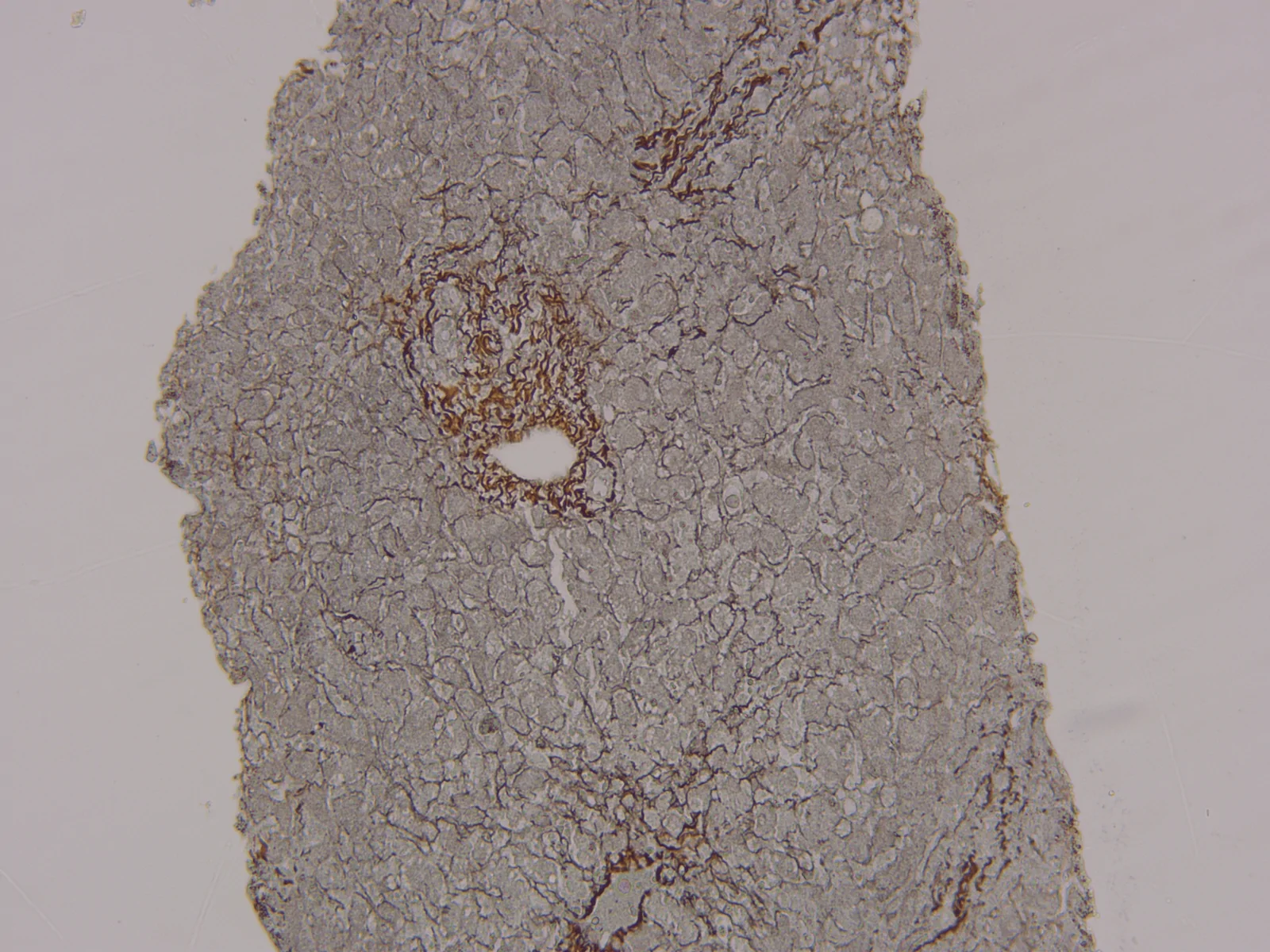
Reticulin Staining X10. Preserved architecture of the liver.

Biopsy showed preserved liver architecture. On H&E-stained sections (Figure 1), the main histological findings were intralobular mixed mononuclear inflammatory infiltration and intracellular and canalicular cholestasis. There was no expansion of the portal tracts by lymphoplasmacytic infiltration. No plasma cell clusters were identified. Masson's trichrome (Figure 2) and reticulin stains (Figure 3) showed no evidence of fibrosis and preserved architecture of the liver. PAS, Perls, and Wilson stains revealed no significant abnormalities.

After biopsy, the consultant hepatologist of the infectious disease clinic recommended starting 32 milligrams of methylprednisolone for suspected idiopathic autoimmune hepatitis, and after two weeks of induction therapy, continuing with azathioprine. The patient was discharged from the clinic with a preliminary diagnosis of idiopathic autoimmune hepatitis (AIH score: 7).

The patient subsequently presented to our clinic for a second opinion about the diagnosis (Table 2). On physical examination, the patient appeared anicteric. No skin rash or lymphadenopathy was observed. Abdominal examination revealed no tenderness, guarding, or rebound tenderness. There was no hepatosplenomegaly.

**Table 2:** Follow-up Laboratory Findings After Approximately Two Weeks of Methylprednisolone Therapy (Presentation Day to Our Clinic) AST, aspartate aminotransferase; ALT, alanine aminotransferase; U/L, units per liter; µmol/L, micromoles per liter.

Parameter	Result	Reference range
Aspartate aminotransferase (AST), U/L	306.53	10-40
Alanine aminotransferase (ALT), U/L	51.02	7-56
Total bilirubin, µmol/L	296.98	3-21
Direct bilirubin, µmol/L	103.97	0-7

Based on the histopathological findings of the liver biopsy, which were not compatible with idiopathic autoimmune hepatitis, and the history of association with albendazole exposure, we suspected drug-induced liver injury mimicking autoimmune hepatitis (RUCAM-8) rather than idiopathic autoimmune hepatitis.

We recommended gradual tapering and discontinuation of corticosteroid therapy and not starting prescribed azathioprine therapy with close biochemical follow-up. If there is a recurrence of transaminase elevation after corticosteroid withdrawal, we would suggest idiopathic underlying autoimmune hepatitis. On the other hand, if the liver tests stay normal without any relapse during follow-up, the liver injury was likely caused by the albendazole medication. The patient was also prescribed ursodeoxycholic acid for continued cholestasis, and we recommended repeat lab tests after five days.

**Figure 4:**
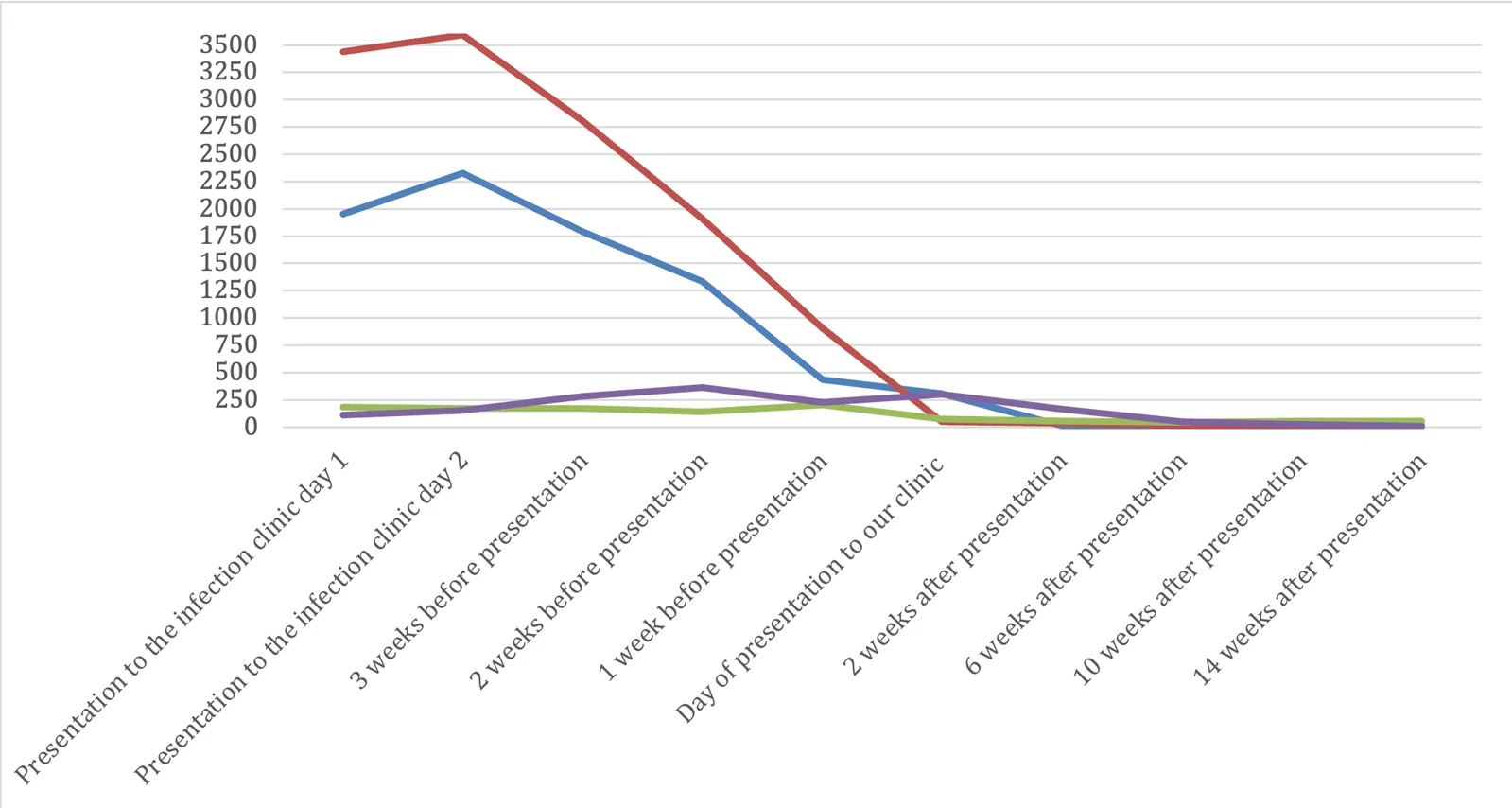
Temporal Trends in Laboratory Parameters Before and After Presentation to Our Clinic

Subsequent follow-up laboratory evaluations after 14 weeks of presentation demonstrated complete normalization of liver biochemistry, with persistently improving bilirubin levels and liver function tests (Table 3, Figure 4).

**Table 3:** Serial Laboratory Findings During Follow-up AST, aspartate aminotransferase; ALT, alanine aminotransferase; ALP, alkaline phosphatase; INR, international normalized ratio; PT, prothrombin time; IgG, immunoglobulin G; ANA, antinuclear antibody; U/L, units per liter; µmol/L, micromoles per liter; g/L, grams per liter; sec, seconds.

Time point	AST, U/L (10–40)	ALT, U/L (7–56)	ALP, U/L (40–130)	Total bilirubin, µmol/L (3–21)	Direct bilirubin, µmol/L (0–7)	INR (0.8–1.2)	PT, sec (11–14)	IgG, g/L (5.52–16.31)	ANA, titer (reference: negative)
Presentation to the infectious diseases clinic, day 1	1,950.00	3,440.00	183	108	74.5	1.51	20.8	22.44	Positive (1:320)
Presentation to the infectious diseases clinic, day 2	2,325.00	3,594.00	170	151.5	—	—	—	—	—
Three weeks before presentation to our clinic	1,794.00	2,810.00	170	284.4	—	—	—	—	—
Two weeks before presentation to our clinic	1,336.00	1,908.00	141	362.5	—	—	—	—	—
One week before presentation to our clinic	433	908	202	225.7	—	—	—	—	—
Day of presentation to our clinic	306.53	51.02	72	296.98	103.97	—	—	—	—
Two weeks after presentation	13.92	34.75	54.78	162.53	51.19	0.89	12.3	11.45	—
Six weeks after presentation	15	15	47	50.7	—	—	—	—	—
10 weeks after presentation	13	11	52	25.3	—	—	—	—	—
14 weeks after presentation	16	15	53	9	3	—	—	9	—

## Discussion

DILI is still a major diagnostic challenge as it has a broad clinical spectrum and the ability to closely mimic other liver diseases, including idiopathic autoimmune hepatitis. Drug-induced autoimmune hepatitis is similar to a recognized phenotype of DILI characterized by hepatocellular injury, positive autoantibodies, elevated immunoglobulin G (IgG) levels, and histopathological features mimicking idiopathic autoimmune hepatitis. Differentiating between these two diagnoses is important because long-term prognosis and management strategies differ [4].

The mechanism of drug-induced autoimmune hepatitis remains incompletely understood. Current evidence suggests that taking certain medications may trigger immune-mediated hepatic injury through the formation of reactive metabolites that interact with hepatocellular proteins. Reactive metabolites generated from hepatic metabolism of drugs bind to cellular proteins such as components of CYP450, which are then recognized as neoantigens by a heightened immunological response, leading to idiopathic autoimmune hepatitis as a result of a misdirected immune response against self [5]. In patients who also have a genetic predisposition, this process may promote loss of immune tolerance and activation of lymphocytes, leading to liver injury. As a result, patients may develop clinical and histopathological findings that are closely similar to idiopathic autoimmune hepatitis.

In our case, the patient developed severe hepatocellular liver injury (R score: 43.6) shortly after albendazole exposure, with markedly elevated aminotransferases, hyperbilirubinemia, positive ANA titers, and elevated serum IgG levels. These findings initially supported a diagnosis of idiopathic autoimmune hepatitis (AIH score: 7) and led to the start of corticosteroid therapy. However, further evaluations later suggested a diagnosis of albendazole-induced DILI mimicking autoimmune hepatitis (RUCAM-8) rather than idiopathic autoimmune hepatitis. Additionally, the patient developed a fever, which can be interpretation of immune allergic reaction [6].

Usually, albendazole is thought to be safe, but sometimes it can cause serious liver damage. This can include hepatocellular injury, cholestatic hepatitis, and autoimmune-like liver injury. The latency period observed in our patient was compatible with previously reported cases of albendazole-induced hepatotoxicity [7].

There is a clear link between taking albendazole and the onset of symptoms. The patient developed symptoms after two weeks of albendazole exposure, which matches the timeline described in LiverTox, making albendazole-induced DILI mimicking autoimmune hepatitis more likely than idiopathic autoimmune hepatitis [2].

Despite rapid improvement in aminotransferase levels after corticosteroid initiation, the patient demonstrated persistent hyperbilirubinemia. It also raised concerns that the underlying process might not be idiopathic autoimmune hepatitis. After reducing the patient's steroid dose and starting ursodeoxycholic acid, liver enzymes, bilirubin, and other blood tests all went back to normal. The subsequent normalization of liver enzymes, bilirubin, coagulation parameters, and serum IgG levels during steroid tapering without evidence of relapse strongly supported a drug-induced process rather than idiopathic autoimmune hepatitis, which typically demonstrates relapse after withdrawal of immunosuppressive therapy.

The presence of positive antibodies such as ANA is frequently associated with DILI, which is well recognized and does not necessarily establish the diagnosis of idiopathic autoimmune hepatitis. Autoantibodies are frequently present in DILI, regardless of the causative drug. Several medications have been associated with autoimmune-like DILI, including nitrofurantoin, minocycline, methyldopa, hydralazine, and, less commonly, albendazole [3].

In such cases, liver biopsy and longitudinal follow-up are often essential for accurate diagnosis. The absence of recurrent hepatitis after corticosteroid withdrawal is considered one of the most important distinguishing features favoring drug-induced autoimmune hepatitis over idiopathic autoimmune hepatitis. The hepatotoxicity of albendazole may be underestimated, given the asymptomatic liver damage, most of which may be anicteric. We recommended that the patient avoid subsequent exposure to Albendazole, as the next time it may cause more severe liver injury.

This case highlights the importance of careful medication history assessment in patients presenting with acute hepatitis and autoimmune serologies. Premature labeling of patients as having idiopathic autoimmune hepatitis may expose them to unnecessary long-term immunosuppressive therapy. Recognition of DILI mimicking idiopathic autoimmune hepatitis is therefore critical for appropriate management and prognosis.

## Conclusions

We describe a case of DILI presenting with clinical, biochemical, and immunological features mimicking idiopathic autoimmune hepatitis. The diagnosis was supported by the association with albendazole exposure, histopathological evaluation, progressive biochemical recovery, normalization of IgG levels, and sustained biochemical improvement during corticosteroid tapering, without starting azathioprine and further follow-up without relapse. This case emphasizes the importance of considering DILI in the differential diagnosis of acute hepatitis with positive autoimmune markers and highlights the critical role of longitudinal follow-up in distinguishing drug-induced autoimmune hepatitis from idiopathic autoimmune hepatitis.
